# Theta coupling within the medial prefrontal cortex regulates fear extinction and renewal

**DOI:** 10.1016/j.isci.2022.105036

**Published:** 2022-08-31

**Authors:** Cong Wang, Peter G. Stratton, Pankaj Sah, Roger Marek

**Affiliations:** 1 Engineering Research Center of Traditional Chinese Medicine Intelligent Rehabilitation, Ministry of Education, Shanghai, China; 2 Queensland Brain Institute, The University of Queensland, Brisbane, Australia; 3 Australian Research Council Centre of Excellence for Integrative Brain Function, Melbourne, Australia; 4 Joint Center for Neuroscience and Neural Engineering, and Department of Biology, Southern University of Science and Technology, Shenzhen, Guangdong Province, P. R. China, 518055

**Keywords:** Behavioral neuroscience, biological sciences, neuroscience, sensory neuroscience

## Abstract

Fear learning, and its extinction, are fundamental learning processes that allow for a response adaptation to aversive events and threats in the environment. Thus, it is critical to understand the neural mechanism that underpins fear learning and its relapse following extinction. The neural dynamics within the subregions of the medial prefrontal cortex, including the prelimbic cortex (PL) and the infralimbic (IL) cortex, and functional connectivity between them during fear extinction and its relapse, are not well understood. Using *in-vivo* electrophysiological recordings in awake behaving rats, we identified increased theta activity in the PL during fear learning and in the IL following extinction. Importantly, the PL-IL theta coupling is significantly enhanced throughout fear learning and extinction, but not in fear relapse. Together, our results provide evidence for the importance of synchronized PL-IL activity to regulate context-dependent retrieval of a fear extinction memory.

## Highlights

• CS + -evoked theta local field potentials (LFPs) power enhancement in fear acquisition is specific to the PL
• Late extinction causes enhanced theta LFP power in the IL, but not PL
• Fear renewal causes enhanced PL and reduced IL theta LFP activity
• PL-IL theta coupling is significantly reduced during fear relapse

## Graphical abstract

**Figure:**
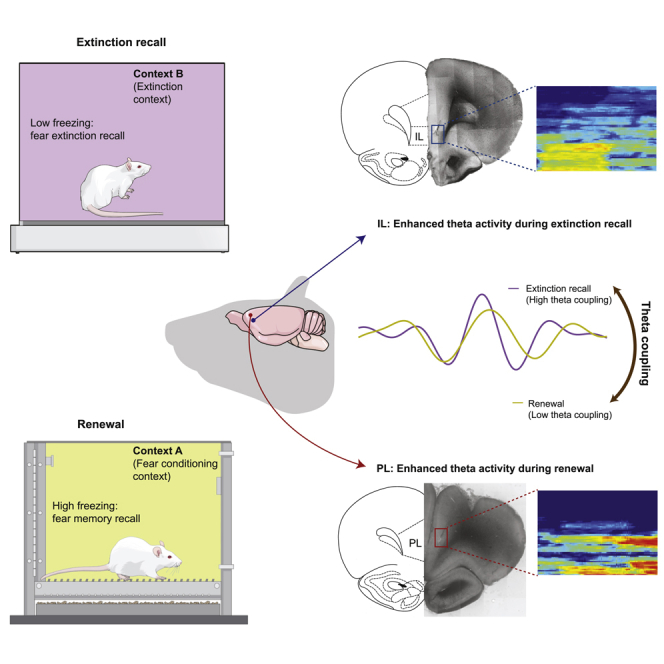

## Introduction

Fear is an evolutionally conserved response, which allows humans and animals to recognize danger and shape adaptive behavior in a rapidly changing environment. However, maladaptive fear can be pathological, leading to disorders such as phobia and post-traumatic stress. In contrast to innate fear, learned fear results from an encounter with threat in the wild, or is experimentally induced by pairing an aversive unconditioned stimulus (US) with a neutral conditioned stimulus or setting/context (CS) to elicit a conditioned response (CR). Even though a fear memory is very robust, the fear response can be extinguished through repeated presentations of the CS alone through a context-specific learning process called extinction (Delamater, 2004). Extinction serves as the basis for exposure-based therapy to treat a variety of anxiety-related disorders (Paredes and Morilak, 2019). However, this process only transiently suppresses learned fear, and is highly context-dependent, often causing the fear to relapse outside the extinction context (Bouton and Bolles, 1979).

While the amygdala is a key structure for fear processing (Sah et al., 2003), the medial prefrontal cortex (mPFC) has been identified to critically regulate fear responses (Marek et al., 2013; Giustino and Maren, 2015). More specifically, the prelimbic cortex (PL) has been shown to predominantly drive fear expression (Burgos-Robles et al., 2009; Corcoran and Quirk, 2007; Kim et al., 2013; Sharpe and Killcross, 2015), whereas the infralimbic cortex (IL) regulates the extinction of learned fear (Milad and Quirk, 2002; Quirk and Mueller, 2008; Sierra-Mercado et al., 2011; Vidal-Gonzalez et al., 2006; Lebron et al., 2004). However, this idea of the segregated function of the PL and IL has recently been challenged as direct synaptic inter-connectivity between these regions affects the extinction of fear (Marek et al., 2018; Mukherjee and Caroni, 2018) and cocaine seeking (Visser et al., 2022). A wealth of research has explored the roles of individual mPFC subregions at specific time points during fear and extinction, but the nature of network activity within the PL and IL during fear learning, expression, extinction, and fear relapse are not fully understood. In the current study, local field potentials (LFPs), that provide a direct readout of neural population activity over time (Einevoll et al., 2013; Buzsáki, 2006; Buzsaki et al., 2012), were simultaneously recorded in the PL and IL throughout the course of fear acquisition, extinction, and recall within and outside the extinction context (renewal). We characterized changes in theta (4–10 Hz), slow gamma (30–55 Hz), and fast gamma (55–100 Hz) band LFP oscillations (Buzsaki and Draguhn, 2004) evoked by the conditioned stimulus (CS+) throughout the behavioral training. To control for the specificity of the LFP response to the CS+, we used discriminative auditory fear conditioning (DAFC), in which a neutral tone (CS-), which was never paired with a shock, was also presented. Our results provide evidence for a dynamic neural activity pattern within the PL and IL for different stages of fear and extinction. CS + -evoked theta activity in the PL was found to be increased in high fear states (fear acquisition and relapse), whereas, in the IL, theta power increased in late extinction learning but decreased during fear renewal. Moreover, CS + -evoked theta oscillations in the PL and IL activity were synchronized throughout fear-related learning (fear acquisition and extinction), but reduced during fear relapse.

## Results

### Conditioned stimulus + -evoked theta local field potentials power enhancement after fear acquisition is specific to the prelimbic cortex

**Figure 1:**
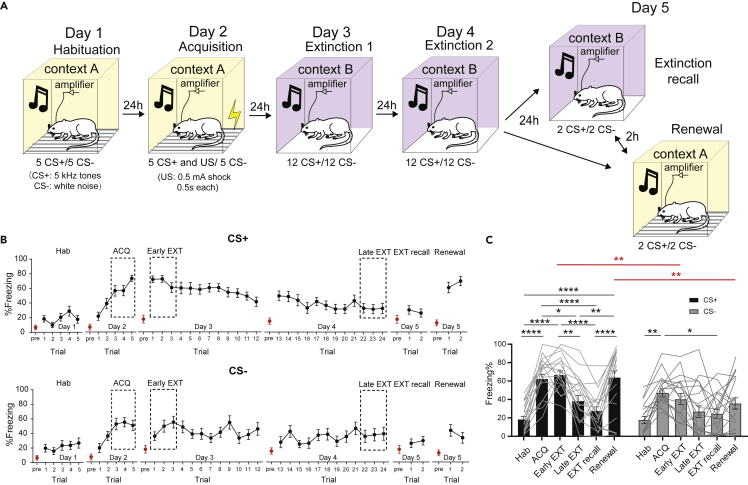
Fear response to the CS+ and CS- in a DAFC task (A) Schematic of the 5-day discriminative auditory fear conditioning protocol. Yellow color, context A; purple color, context B. (B) Freezing level of the trial by trial CS+ (top) and CS- (bottom) presentation during the 5-day DAFC task. Dotted rectangle frames show the %freezing values used for quantification. Red dots, pre-tone baseline freezing behavior; black dots, trial freezing behavior. (C) Rats showed a significant difference in freezing response from the CS+ and CS- during the ACQ and Renewal sessions (Two-way ANOVA with multiple comparisons, row factor *F*(5,227) = 17.46, p < 0.0001, column factor *F*(1,227) = 22.81, p < 0.0001. ∗p < 0.05, ∗∗p < 0.01, ∗∗∗p < 0.001, ∗∗∗∗p < 0.0001. All the N = 20 rats). Graphs show the mean freezing percentage ± s.e.m.

All experimental rats were implanted with movable electrodes into the PL and IL (see STAR Methods). Freezing remained low before CS onset, (Figure 1B, red dots), showing that the behavioral response to the CS presentation is entirely cue-induced. On Day 1, animals were habituated to context A with 5 CS+ (6 kHz) and CS- (white noise) presented in a pseudorandom order and showed low levels of freezing to both. On Day 2, rats underwent DAFC in the same context with a pseudorandom order of 5 CS + paired with the US and 5 unpaired CS- (Figure 1A). The animals gradually developed a CR to the CS + as the freezing percentage increased from trial 1 to trial 5, reaching significantly higher levels by the end of the fear acquisition and fear expression session (Figure 1B, top; ACQ: trials 3–5; Early EXT: trial 1–3) compared to the habituation session (Figure 1C, two-way ANOVA with multiple comparisons, Hab v.s. ACQ, Hab v.s. Early EXT, ∗∗∗∗p < 0.0001, N = 20). However, we did not observe any significant difference in the freezing behavior in early (0–10 s), middle (10–20 s), or late (20–30 s) stages of the CS + presentation during the ACQ session or Early EXT session (Figure S1). In contrast, fear responses to the CS- remained significantly lower compared to the CS+ during the early extinction (fear expression) session (Figure 1C, two-way ANOVA with multiple comparisons, ACQ CS + v.s. ACQ CS-, ∗∗p < 0.01, N = 20).

**Figure 2:**
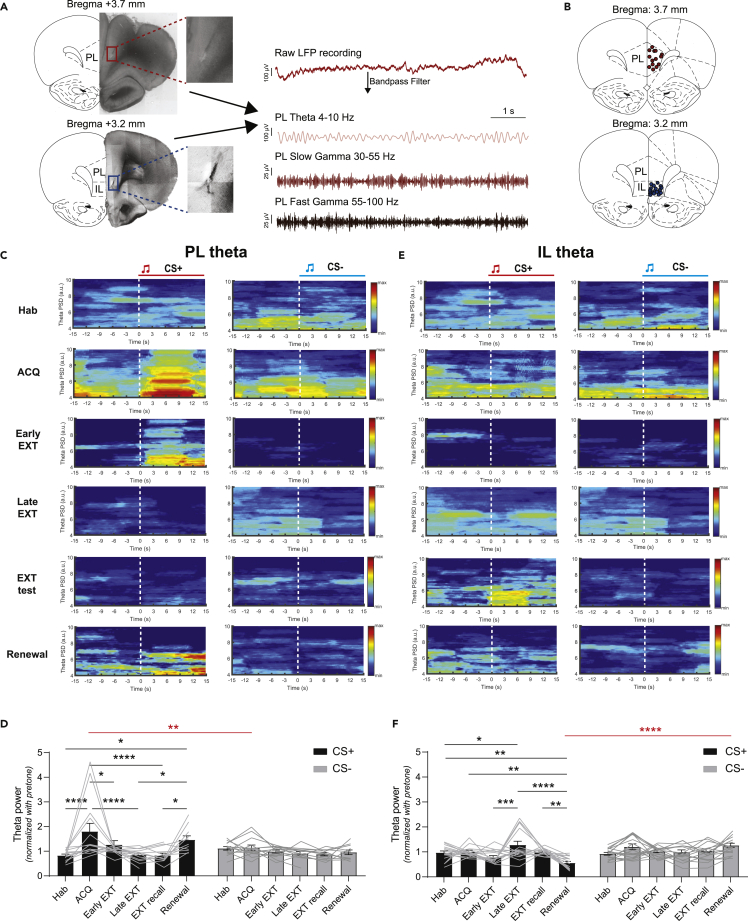
Subregion-specific theta activity in the mPFC to the CS+, but not CS- during DAFC (A) Illustration of LFPs simultaneously recorded from the PL (left) and IL (right) in rats performing the DAFC task. The PL and IL LFPs were filtered into theta 4–10 Hz, slow gamma 30–55 Hz, and fast gamma 55–100 Hz frequency bands for analysis (gamma activity data shown in Figure 3). (B) Placement of electrode tips in the PL (top, n = 14) and IL (bottom, n = 18) for all the rats performing the DAFC task. (C) One representative trial of the PL theta LFP (4–10 Hz) power spectral density during the CS+ (left panel)/CS- (right panel) presentation in the DAFC task. Dashed white lines represent CS+/CS- onsets. (D) Mean normalized PL theta power (4–10 Hz) during the CS + presentation or (E) CS- presentation in different sessions of the DAFC task (Two-way ANOVA with mixed effects model, *F*(5,117) = 6.558558, p < 0.0001.. ∗p < 0.05, ∗∗p < 0.01, ∗∗∗p < 0.001, ∗∗∗∗p < 0.0001, n = 14 LFP recording sites). (E) One representative trial of the IL theta LFP (4–10 Hz) power spectral density during the CS+ (left panel)/CS- (right panel) presentation in the DAFC task. Dashed white lines represent CS+/CS- onsets. (F) Mean normalized IL theta power (4–10 Hz) ± s.e.m. during the CS + presentation or (H) CS- presentation in different sessions of the DAFC task (Two-way ANOVA with mixed effects model, *F*(5,144) = 3.226226, p < 0.01. ∗p < 0.05, ∗∗p < 0.01, ∗∗∗p < 0.001, ∗∗∗∗p < 0.0001, n = 18 LFP recording sites).

**Figure 3:**
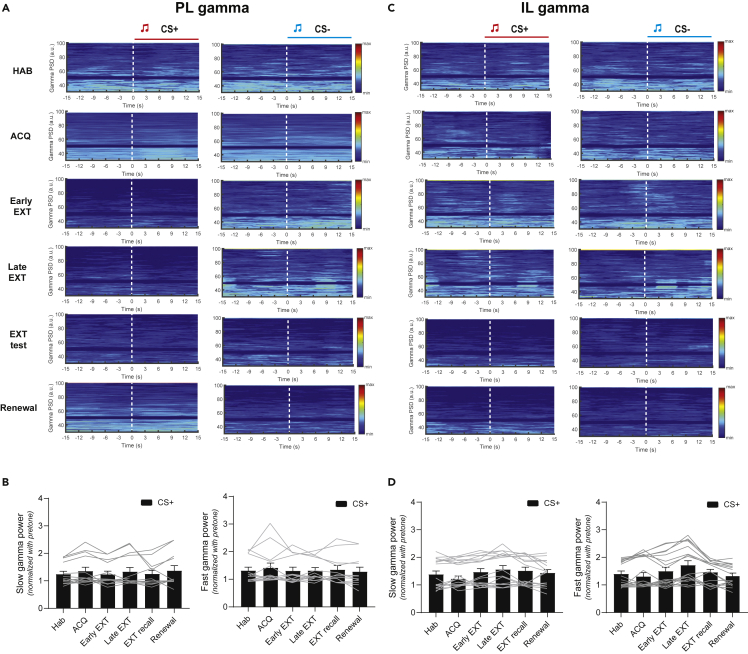
There is no change in CS + -evoked slow or fast gamma activity in the PL and IL during DAFC (A) One representative trial of the PL gamma LFP (30–100 Hz) power spectral density during the CS+ (left panel)/CS- (right panel) presentation in the DAFC task. Dashed white lines represent CS+/CS- onsets. (B) Mean normalized PL slow gamma (30–55 Hz, left panel) or fast gamma (55–100 Hz, right panel) power ± s.e.m. during the CS + presentation in different sessions of the DAFC task. p > 0.05, n = 14 LFP recording sites). (C) One representative trial of the IL gamma LFP (30–100 Hz) power spectral density during the CS+ (left panel)/CS- (right panel) presentation in the DAFC task. Dashed white lines represent CS+/CS- onsets. (D) Mean normalized IL slow gamma (30–55 Hz, left panel) or fast gamma (55–100 Hz, right panel) power ±s.e.m. during the CS + presentation in different sessions of the DAFC task. (Ordinary one-way ANOVA with multiple comparisons, all p > 0.05, n = 18 LFP recording sites).

Theta oscillations in the LFP in fear-related brain regions reflect synchronized neural firing, believed to facilitate communication between regions in response to aversive stimuli (Stujenske et al., 2014a; Likhtik et al., 2014). Thus, we sought to investigate theta oscillation in the PL and IL (Figures 2A and 2B) through the course of fear learning and extinction. In line with the fear behavior, CS + -evoked PL theta (4–10 Hz) LFP power was significantly enhanced after fear acquisition compared to the habituation session (Figures 2C and 2D left, two-way ANOVA with mixed effects model, Hab v.s. ACQ, ∗∗∗∗p < 0.0001, n = 14 recording sites). No significant change in PL LFP power was observed during CS- presentations (Figure 2D right, two-way ANOVA with mixed effects model, Hab v.s. ACQ, p > 0.05, n = 14 recording sites). In contrast, CS + -evoked theta LFP power in the IL did not change significantly in fear acquisition for either CS + or CS- (Figures 2E and 2F left, p > 0.05, n = 18 recording sites). Notably, there was no change in CS + -evoked LFP power for slow (30–55 Hz) or fast gamma (55–100 Hz) bands in either PL or IL (Figures 3A–3D). One day after fear acquisition, the fear memory was retained and retrieved (fear expression) in context B (Figure 1A, Day 3, purple). Retrieval of fear memory was assessed by averaging the first block of fear extinction (Figure 1B, Early EXT: trial 1–3). As expected, retrieval of fear memory by the CS + evoked greater freezing as compared to the habituation session (Figure 1C, two-way ANOVA with multiple comparisons, Hab v.s. Early EXT, ∗∗∗∗p < 0.0001, N = 20 rats). Moreover, in both the PL and the IL, there was no change in evoked theta and gamma LFP power by either CS + or CS- during fear retrieval (Figure 2D, 2F, and 3A–3D).

### Late extinction causes enhanced theta local field potential power in the infralimbic, but not prelimbic cortex

Next, rats underwent extinction in context B over two days (Figure 1A, Day 3 and 4) and showed a gradual decline in freezing to CS + presentations (Figure 1B, top), with significantly reduced freezing levels by the end of the second extinction session (Figure 1C left, two-way ANOVA with multiple comparisons, Early EXT v.s. Late EXT, ∗∗p < 0.01, N = 20 rats). The theta activity in the PL exhibited power reduction in the late stage of the extinction learning (Figure 2D left, two-way ANOVA with mixed effects model, Hab v.s. Late EXT, ∗∗∗∗p < 0.0001, n = 14 recoding sites), while theta activity remained low during CS- presentations. Meanwhile, extinction caused enhanced theta LFP power in the IL compared to the habituation or early extinction session (Figure 2D left, with mixed effects model, Hab v.s. Late EXT, ∗p < 0.05, Early EXT v.s. Late EXT, ∗∗∗p < 0.001, n = 18 recoding sites). This CS + -evoked oscillatory activity power change is not observed over the course of extinction in either PL or IL in the slow (30–55 Hz) or fast gamma (55–100 Hz) band (Figures 3A–3D).

### Fear renewal causes enhanced prelimbic and reduced infralimbic theta local field potential activity

On day 5, animals were first tested in the extinction context (EXT recall). Extinction is context-specific (Maren et al., 2013) and to test for renewal of fear, they were returned to context A (Figure 1A, Day 5). Presentation of CS+ in context B (extinction context) evoked low levels of freezing (Figure 1B, top), but then returned to context A, CS + -evoked fear returned (Figure 1B, top; Figure 1C left, two-way ANOVA with multiple comparisons, EXT recall v.s. Renewal, ∗∗∗∗p < 0.0001, N = 20 rats). However, the response to CS- during renewal remained significantly low (Figure 1C, two-way ANOVA with multiple comparisons, Renewal CS + v.s. Renewal CS-, ∗∗p < 0.01, N = 20 rats). Concurrent LFP recordings show that in the PL, CS + -evoked theta power significantly enhanced during renewal compared to the extinction recall session or habituation session (Figures 2C and 2D, two-way ANOVA with mixed effects model, EXT reacall v.s. Renewal, Hab v.s. Renewal, ∗p < 0.05, n = 14 recording sites). In contrast, in the IL, CS + evoked theta power was significantly lower in renewal than during late extinction or extinction recall session (Figure 2F left, two-way ANOVA with mixed effects model, Late EXT v.s. Renewal, ∗∗∗∗p < 0.0001, EXT reacall v.s. Renewal, ∗∗p < 0.01, n = 18 recording sites). These effects were specific to the CS+ (Figure 2E right; Figure 2F right), and were limited to the theta band (Figures 3A–3D).

### Prelimbic-infralimbic theta coupling is significantly reduced during fear relapse

LFP recordings from the mPFC in *ex-vivo* slices have hinted toward synchronized activity between the PL and IL (Van Aerde et al., 2008). We thus investigated coupled activity between the PL and IL across behavioral states using cross-correlation analysis (Adhikari et al., 2010; Wang et al., 2021). This method assumes that functional connectivity between the two structures is accompanied by reasonably coherent activity within a specific frequency range. Therefore, the peak cross-correlation coefficient is used as the indicator for functional connectivity strength.

**Figure 4:**
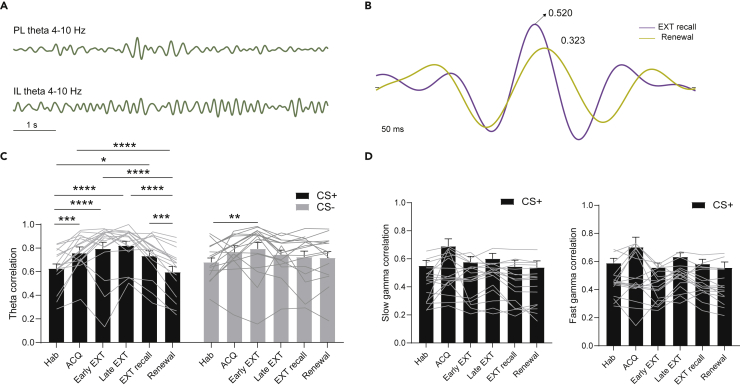
PL-IL theta coupling is significantly enhanced during extinction, but not fear relapse (A) Example of a simultaneously recorded PL (top) and IL (bottom) LFP filtered into the 4–10 Hz theta band. (B) PL-IL theta LFP cross-correlation of one representative rat reduced from the EXT recall (purple line, peak cross-correlation coefficient: 0.520) session to the renewal (yellow line, peak cross-correlation coefficient: 0.323) session. (C) Mean PL-IL theta LFP cross-correlation coefficient ±s.e.m. during the CS + presentation or CS- presentation in different sessions of the DAFC task (Two-way ANOVA with mixed effects model, *F*(5,1432.674,76.47)=14.8181, p < 0.0001. ∗p < 0.05, ∗∗p < 0.01, ∗∗∗p < 0.001, ∗∗∗∗p < 0.0001, n = 17 PL-IL LFP recording pairs). (D) Mean PL-IL slow gamma (30–55 Hz, left) and fast gamma (55–100 Hz, right) LFP cross-correlation coefficient ± s.e.m. during the CS + presentation in different sessions of the DAFC task (Ordinary one-way ANOVA with multiple comparisons, all p > 0.05, n = 17 PL-IL LFP recording pairs).

Our results (Figure 4) revealed a significant increase in PL-IL theta (4–10 Hz) coupling during all stages of fear and extinction learning compared to the habituation during the CS + presentations, but not the CS- presentations (except during early extinction). Moreover, this CS + -induced elevation in theta coupling was significantly reduced outside the extinction context during the renewal (Figure 4C, left, two-way ANOVA with mixed effects model, EXT recall vs. Renewal, ∗∗∗p < 0.001, n = 17 pairs), an effect that was not observed during CS- presentations (Figure 4C, right, two-way ANOVA with mixed effects model, EXT recall vs. Renewal, p > 0.05, n = 17 pairs). This increase in synchronized theta activity between the PL and IL was not reflected in the slow (30–55 Hz) and fast gamma (55–100 Hz) bands (Figure 4D).

## Discussion

The medial prefrontal cortex is well known to have a role in fear expression and fear suppression following extinction learning. Previous studies have largely focused on investigating the role of the PL or IL in fear expression and extinction separately, with very few studies exploring responses to the CS+ and CS- across the whole behavioral span of aversive learning and its extinction. Here, using animals with recording electrodes simultaneously implanted in the PL and IL, we have tested animals from habituation to fear renewal. This has permitted an analysis of rodent behavior as well as the dynamics of neural activity in these two linked subregions of the mPFC that contribute to fear- and extinction learning, its retrieval, and relapse.

Using differential auditory fear conditioning, we specifically focused on examining CS+/CS- evoked mPFC LFP activity in the theta (4–10 Hz), slow gamma (30–55 Hz) and fast gamma (55–100 Hz) range during different behavioral states. As expected, following pairing with the US, rats showed enhanced freezing in response to the CS+, as compared to the unpaired CS-. Notably, some animals also show enhanced freezing to the CS- , which is most likely owing to a generalized fear response (Likhtik et al., 2014). Extinction of this learned fear by repeated presentations of the CS+, in one context, led to a reduced fear response, but the fear returned (relapse) when tested outside the extinction context. These results are in line with Pavlov’s classical findings, which he described as “internal inhibition of conditioned reflexes” (Pavlov and Anrep, 1927). The return of fear after full extinction (24 trials) in the renewal session also supports the theory that extinction is a new form of learning (Bouton, 2004) that is, at least in part, regulated by context exposure.

Recent electrophysiological studies of fear-related behaviors have shown that LFP oscillations are prominently seen in fear-related brain structures such as the amygdala and mPFC (Likhtik et al., 2014; Dejean et al., 2016; Stujenske et al., 2014b). These oscillations are elicited by CS + presentations or freezing behavior, suggesting that they play important roles in processing fear information. In line with a recent study showing that PL LFP activity temporally coincides with CS + -induced freezing (Likhtik et al., 2014), our findings revealed that the CS + -evoked PL theta LFP (4–10 Hz) power significantly increased at the end of fear acquisition (Figure 2D), a state of high fear. However, theta activity in the PL was not altered over the course of extinction learning and its retrieval, which stands in contrast with a previous study by Fenton et al. (2016). This discrepancy might be owing to the dependence on the precise placing of the electrodes and how these regions are interacting with each other (in the present study, recording electrodes are also placed apart in the anterior/posterior plain). However, consistent with previous studies (Fenton et al., 2014, 2016; Milad and Quirk, 2002), LFP theta activity in the IL was enhanced at the end of extinction learning (late extinction). Our study also revealed that IL theta LFP oscillations are significantly reduced during fear renewal (Figure 2F). Surprisingly, no significant enhancement in IL theta LFP power was found in extinction retrieval, suggesting that IL theta oscillations are required for extinction learning. Given that PL theta activity was not significantly changed throughout extinction and retrieval, volume conduction of neural activity across mPFC subregions is likely to be minimal. Furthermore, our results revealed a theta-oscillation mediated neural synchrony between the PL and IL during fear acquisition, extinction, and extinction recall, but not renewal, suggesting a mechanism by which a PL-IL communication is able to facilitate context-specific learning. This coherent activity was specific to the CS+, suggesting that enhanced coupling between the PL and IL is owing to learning-specific retrieval of CS + -related information. Although prior lesioning studies (Laurent and Westbrook, 2009) and single-unit recordings (Milad and Quirk, 2002) suggested that PL activity is not required in extinction, our findings are in agreement with recent studies, indicating that the PL can affect extinction (Marek et al., 2018; Burgos-Robles et al., 2007). Given that PL-IL theta coupling was specifically suppressed during fear relapse, our results suggest that PL-IL synchrony is mainly driving recall of the extinction memory, rather than just facilitating the retrieval of context-dependent information following fear extinction.

Together, our results provide evidence for fear state-dependent neural oscillations with increased PL theta activity in high fear states (fear learning and renewal) and suppressed IL theta oscillations in fear renewal. We have identified a discrepancy in theta coupling between PL and IL during extinction recall versus fear relapse, and thereby challenging the current neural circuity model of distinct roles of the PL and IL in fear and extinction, respectively.

### Limitations of the study

A possible limitation of our study is related to the sex of our experimental animals. We chose male rats for more consistent behavior in the fear conditioning task. Also, it has been reported that the neural oscillatory activity in the prefrontal cortex might be different between female and male rodents (Fenton et al., 2016). Thus, our study aimed to control the sex variable. Another possible limitation is that we have not recorded and measured the single-unit activity in the PL and IL. Therefore, we did not explore the role of the PL-IL interaction during fear extinction and renewal at the cellular level.

## STAR★Methods

### Key resources table

**Table:** 

REAGENT or RESOURCE	SOURCE	IDENTIFIER
**Deposited data**		

The full dataset and custom MATLAB code	This paper	Available upon request

**Experimental models: Organisms/strains**		

Spray Dawley Rats	Animal Resources Centre (Perth Australia)	N.A.

**Software and algorithms**		

MATLAB	MathWorks	version R2016b
Prism	GraphPad	version 8.0
Animal behaviour analysis	Noldus Information Technology	EthoVision XT 13.0

**Other**		

Neural recording system	Axona Ltd., UK	DacqUSB
Animal behaviour video recording	Axona Ltd., UK	Dacqtrack
LFP recording electrode	Neuralynx, USA	Versa2,4 and 8 microdrives

### Resource availability

#### Lead contact

Further information and requests for resources and reagents should be directed to and will be fulfilled by the lead contact, Roger Marek (r.marek@uq.edu.au).

#### Materials availability

This study did not generate new unique reagents.

### Experimental model and subject details

Male Sprague-Dawley rats (5–6 weeks, 220g–350g) were obtained from the Animal Resources Centre (Perth, Australia) and housed in groups of 2–4 in OptiRAT cages or standard cages under a 12 h light-dark cycle (light phase: 7 a.m.-7 p.m.), with food and water provided *ad libitum*. All experiments were performed during the light phase. All procedures were performed in accordance with the Australian Code for the Care and Use of Animals for Scientific Purposes and the Australian Code for the Responsible Conduct of Research and approved by the Animal Ethics Committee at the University of Queensland.

### Method details

#### VersaDrive construction

VersaDrives with two or four independently movable drives were assembled according to the VersaDrive construction manual (Neuralynx, Bozeman, MT). Each wire of the independent electrodes as well as 2 ground wires were stripped of insulation at the end and connected to a gold-plated receptacle. Following assembly of the VersaDrive, a plating procedure was performed on each drive’s electrode to reduce the impedance and to enhance the signal to noise ratio (SNR). First, the electrode tips were soaked in the platinum black plating solution (Neuralynx, Bozeman, MT). Next a platinum bundle was used as the anode and a −0.1 μA current was provided on each channel for 20–60 s to form a platinum layer on each tip in order to reduce the impedance to 20–100 kΩ. Ultrasonication (50 W, 40 kHz) was applied during the plating process to remove the weakly bonded plating and to create a stable impedance.

#### Stereotaxic surgery

Stereotaxic surgery was conducted to implant the VersaDrives unilaterally into the mPFC (including the PL and IL). Rats were anaesthetized using isoflurane in air (1.5 %–3%) and were fixed in a stereotaxic frame using the incisor and nonpuncture ear bars (ASI Instruments, Warren, MI). After exposing the skull, a craniotomy was performed and the VersaDrive was implanted aiming at the following coordinates (anterior/posterior, medial/lateral, dorsal/ventral referenced to bregma): PL (+3.7 mm, 0.1–0.9 mm, −3.2 mm); IL (+3.0 mm, 0.1–0.9 mm, −4.8 mm). The ground wires were placed beneath the skull and above the dura in the contralateral side of the skull. Three to four bone screws (Fine Science Tools, North Vancouver, Canada) were anchored in the skull and implants were secured using Super-Bond cement (Sun Medical, Shiga, Japan). The incision in the scalp was sutured, and the edge of skin was attached to dental cement using Vetbond veterinary tissue adhesive (3M, Maplewood, MN). Body temperature was maintained at 37°C with a heating pad during the surgery. Baytril (0.1 μL/g, Bayer, Leverkusen, Germany) and Metacam (0.4 μL/g, Boehringer Ingelheim Vetmedica, Ingelheim am Rhein, Germany) were each diluted into 0.5 mL of saline and then injected subcutaneously at the end of the surgery. After surgery, rats were injected with antibiotic (Baytril, 0.05–0.1 μL/g) for 5 days and housed individually in open plastic cages or standard cages for at least 7 days with food and water provided *ad libitum* and were also habituated to handling.

#### Apparatus

Two distinct chambers with open tops were used as context A and B. Context A: square silver cube cage with metal-grid ground, scent marked by amyl acetate; Context B: round yellow plastic bucket without metal-grid ground, scent marked by acetic acid. Before and after each session, the cages were cleaned with 70% ethanol. The total duration of the CS was 30 s consisting of 500 ms pips (sound pressure level: 75 dB) at 1.5 Hz repeated 20 times. The frequency of the CS pips was 6 kHz for the CS+ and white noise for the CS-. The unconditioned stimulus (US) was a 0.5 mA (0.5 s) foot-shock. Within the CS + -US pair, the onset of the US started at the offset of the last CS + pip. The delays between stimulus presentations were pseudorandom in all training sessions with the inter-trial intervals of 90–120 s.

#### Behavioural protocol

The 5-day discriminative auditory fear conditioning protocol is illustrated in Figure 1A. Animals were allowed to explore the context freely for 3 min before each session to familiarize with the environment and set-up. Day 1 habituation (Hab): animals were habituated in context A and received pseudorandom presentations of 5 CS+ and 5 CS-. Day 2 Acquisition: animals received a pseudorandom order of 5 presentations of the CS + that co-terminated with the US, and 5 presentations of CS- in context A. The last 3 CS presentations were used to evaluate fear learning (ACQ). Day 3 and day 4 extinction (EXT): Animals were exposed to context B and received 12 presentations of CS+ and 12 presentations of CS- in a random order on each day. The first 3 CS presentations on day 3 were used to evaluate fear expression (early EXT) and the last 3 trials of CS presentations on day 4 were used to evaluate extinction learning (late EXT). Day 5 Test: extinction memory recall (EXT test) was first tested in context B with 2 CS+ and 2 CS- presentations. Then the animals were transported to their home cage and rested for at least 2 h. Next, they were placed in context A to test fear memory relapse (Renewal) receiving 2 CS+ and 2 CS- presentations. Animal behaviour was recorded by a digital camera (FlyCapture Flea 2, Point Grey Research, Richmond, Canada) and freezing behaviour was counted and scored manually by the observer. Freezing was defined as the absence of any movement except breathing and was scored if there was no movement for at least 1 s (except sleeping).

#### Signal recording

The implanted VersaDrive was connected to a headstage containing unity-gain operational amplifiers (Axona, St Albans, UK). Each headstage was connected to a 16-channel preamplifier and the recorded primary signals were digitized by the Analog to Digital Converter at a rate of 48 kHz. The digitalised signal was then fed into the system unit and the processing to record LFP was set as a gain of 500× or 1000× with a cut-off filter of 1 kHz and a notch filter of 50 Hz. At the conclusion of the experiment, recording sites were marked with electrolytic lesions before perfusion, and electrode tip locations were reconstructed with standard histological techniques (see details in histology method part). Rats were excluded from LFP data analysis if the electrode placement was outside of the targets.

#### Local field potential analysis

All analyses were done using MATLAB (MathWorks, Natick, MA). Raw LFP signals were clustered into canonical frequency bands: theta (4–10 Hz), slow gamma (30–55 Hz), fast gamma (55–100 Hz) using a discrete-form FIR bandpass filter with characteristics of linear phase response and stability. The attenuation in the stop band was set as 80 dB and the amount of ripple allowed in the pass band was 1 dB. The frequency differences between the start of the 1st stop band and the start of the 1st pass band and between the start of the 2nd pass band and the start of the 2nd stop band were both 0.5 Hz. Data were excluded from LFP data analysis if the signal was contaminated by artefact (e.g.: introduced by the headstage hitting the arena wall or animal grooming).

A 16-times averaged spectrum of the first 15s of the CS presentation (*PSD*_15s CS_) and the 15 s before CS onset (*PSD*_15s pre-CS_) were calculated using the pwelch function (MATLAB) with a Hanning window of sample length/16 points with no overlap. To get the normalized (with pre-CS period) CS-evoked LFP power, the summed PSD during CS presentation was divided by the summed pre-CS PSD and averaged across specific trials in each behavioural session:

$$CS-evoked\;LFP\;power=\frac{\bar{\sum PSD_{15\;s\;CS}}}{\bar{\sum PSD_{15\;s\;pre-CS}}}$$

To determine the functional connectivity (neural coupling) between two brain regions, cross-correlation analysis was performed in MATLAB (xcorr function). The instantaneous amplitudes of the recorded LFPs from the PL and the IL were considered as two discrete-time sequences x(n) and y(n) respectively. The cross-correlation coefficient ${\hat{R}}_{xy,coeff}$ was calculated by the xcorr function in MATLAB as the following formula (the asterisk denotes complex conjugation):

$${\hat{R}}_{xy}\left(m\right)=\left\{ \begin{array}{l} \sum_{n=0}^{N-m-1}x_{n+m}y_{n}^{*},\;m\geq 0,\\ {{\hat{R}}^{*}}_{yx}\left(-m\right),\;m<0. \end{array}\right.$$

$${\hat{R}}_{xy,coeff}\left(m\right)=\frac{1}{\sqrt{{\hat{R}}_{xx}\left(0\right){\hat{R}}_{yy}\left(0\right)}}{\hat{R}}_{xy}\left(m\right)$$

The averaged peak cross-correlation coefficient in different sessions was retrieved by averaging the peak cross-correlation coefficient ${\hat{R}}_{xy,coeff-peak}$ across all the animals during the particular session.

#### Immunohistochemistry

Following behavioural procedures, rats were euthanized with a lethal dose of isoflurane. Electrolytic lesions to mark the tips of the tetrode were generated by a 70 μA direct current for 5 s. Animals were then perfused through the left ventricle with 4% paraformaldehyde in 1 × Phosphate-buffered saline (PBS). Brains were dissected from the skull and fixed in PFA for 24 h at 4°C. Brains were then coronally sliced into 100 μm sections using a vibratome (Leica Biosystems, Wetzlar, Germany). Sections were mounted on microscope slides and coated with antifade solution and a glass coverslip. Electrolytic lesions were identified and verified with an upright fluorescent microscope (Zeiss Axio Imager/Observer, Jena, Germany) and images were taken with a 5× objective using bright field imaging.

### Quantification and statistical analysis

#### Statistical analysis

Prism version 8.0 (GraphPad, San Diego, CA) was used for statistical analysis. Normal distribution was tested in all the dataset using the Kolmogorov-Smirnov test: if the dataset passed the normality test, parametric tests were used. Otherwise, non-parametric tests were used. N represents the number of the animals, and n the number of the recording sites or LFP pairs. Multiple-group comparisons were assessed using one-way ANOVA test with repeated measures followed by a post hoc Tukey’s multiple-comparison test to identify significant groups as indicated in the figure legend. The CS+/CS- presentation and different behavioural sessions were used as two main factors for the two-way ANOVA. Multiple-variate comparisons were assessed using two-way ANOVA with repeated measures or mixed effects model, followed by a post hoc Tukey’s multiple-comparison test to identify significant groups as indicated in the figure legend.

## Data Availability

• The original data reported in this paper is available from the lead contact upon request. • All original code is available from the lead contact upon request. • Any additional information required to reanalyse the data reported in this paper is available from the lead contact upon request.
