# Supplementary material for: Theta coupling within the medial prefrontal cortex regulates fear extinction and renewal
Source: iScience. 2022 Aug 31;25(10):105036. doi: 10.1016/j.isci.2022.105036 (PMC9485106; doi:10.1016/j.isci.2022.105036)
Supplement: Document S1. Figure S1 [file mmc1.pdf]

iScience, Volume *25*

Supplemental information

Theta coupling within the medial prefrontal cortex regulates fear extinction and renewal

Cong Wang, Peter G. Stratton, Pankaj Sah, and Roger Marek

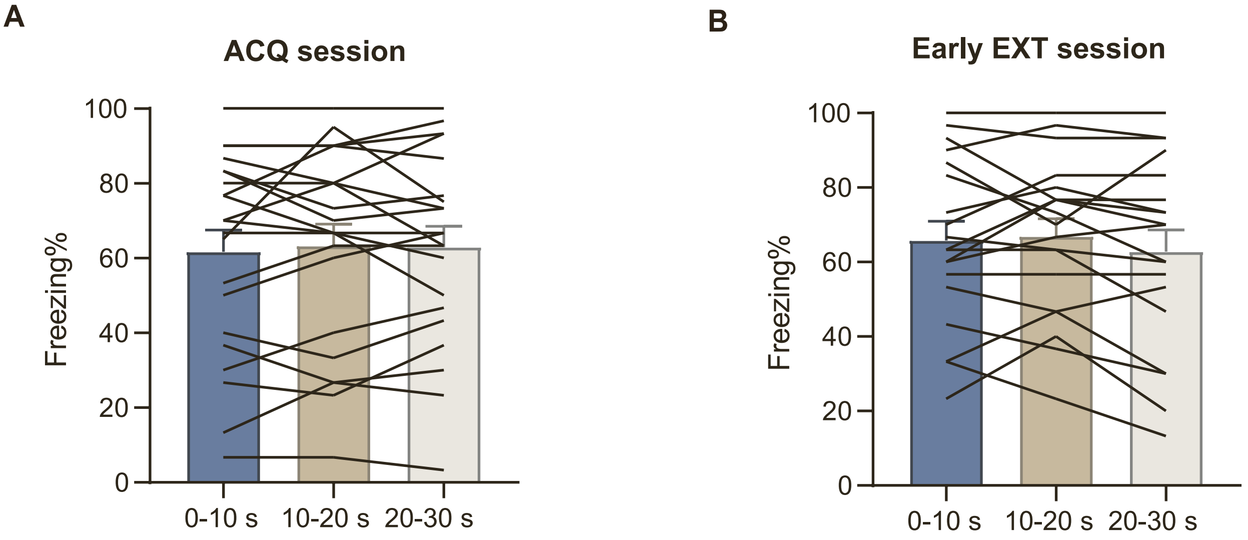

Figure S1. Changes in the freezing level during the early, middle and late stage of the CS+ presentation, related to Figure 1.

(A) The freezing level of the ACQ session and (B) Early EXT session during the first, second and third 10 s of the CS+ presentation (Ordinary one-way ANOVA with multiple comparisons, all $P>0.05$, both $N=19$ rats).
